# EDGE^3^: A web-based solution for management and analysis of Agilent two color microarray experiments

**DOI:** 10.1186/1471-2105-10-280

**Published:** 2009-09-04

**Authors:** Aaron L Vollrath, Adam A Smith, Mark Craven, Christopher A Bradfield

**Affiliations:** 1 McArdle Laboratory for Cancer Research, University of Wisconsin School of Medicine and Public Health, Madison, WI 53706, USA; 2 Department of Biostatistics and Medical Informatics, University of Wisconsin-Madison, Madison, 53706, USA; 3 Department of Computer Science, University of Wisconsin-Madison, Madison, WI 53706, USA

## Abstract

**Background:**

The ability to generate transcriptional data on the scale of entire genomes has been a boon both in the improvement of biological understanding and in the amount of data generated. The latter, the amount of data generated, has implications when it comes to effective storage, analysis and sharing of these data. A number of software tools have been developed to store, analyze, and share microarray data. However, a majority of these tools do not offer all of these features nor do they specifically target the commonly used two color Agilent DNA microarray platform. Thus, the motivating factor for the development of EDGE^3 ^was to incorporate the storage, analysis and sharing of microarray data in a manner that would provide a means for research groups to collaborate on Agilent-based microarray experiments without a large investment in software-related expenditures or extensive training of end-users.

**Results:**

EDGE^3 ^has been developed with two major functions in mind. The first function is to provide a workflow process for the generation of microarray data by a research laboratory or a microarray facility. The second is to store, analyze, and share microarray data in a manner that doesn't require complicated software. To satisfy the first function, EDGE^3 ^has been developed as a means to establish a well defined experimental workflow and information system for microarray generation. To satisfy the second function, the software application utilized as the user interface of EDGE^3 ^is a web browser. Within the web browser, a user is able to access the entire functionality, including, but not limited to, the ability to perform a number of bioinformatics based analyses, collaborate between research groups through a user-based security model, and access to the raw data files and quality control files generated by the software used to extract the signals from an array image.

**Conclusion:**

Here, we present EDGE^3^, an open-source, web-based application that allows for the storage, analysis, and controlled sharing of transcription-based microarray data generated on the Agilent DNA platform. In addition, EDGE^3 ^provides a means for managing RNA samples and arrays during the hybridization process. EDGE^3 ^is freely available for download at .

## Background

The generation of high-density data from microarrays designed to measure transcriptional changes on a whole-genome scale has significantly changed the landscape of biological-based research. Experiments based on microarray technology are now commonplace in private sector, government and academic laboratories. This has been of great benefit to our understanding of biological systems but has introduced some problems related to the use and sharing of high density data. With microarrays the number of processing and quality assurance steps to be documented has increased and the amount of data to be stored, analyzed, and shared has increased exponentially. If microarray experiments are performed on a scale larger than just a few arrays, the need for computational-based solutions to allow for the effective documentation, storage, analysis and sharing of the data generated becomes necessary.

A number of commercial and non-commercial software solutions have been developed to address the problems associated with the scale of microarray data. Some commercial options available to address the aforementioned problems of microarray data include GeneSpring GX[1], Rosetta Resolver[2], Nexus Expression[3], Partek[4], Spotfire[5], and GeneSifter[6]. For some researchers, these commercial options offer an excellent solution to their specific needs. However, a majority of these solutions do not address all of the problems mentioned in relation to the magnitude of microarray data and, if they do, they are, for some, cost prohibitive. Additionally, as commercial software options they may have strict limitations on the number of users who can access the software and the software may only be available on certain operating systems. Non-commercial options available include CARMAweb[7], MAGMA[8], GEPAS[9], Asterias[10], ArrayPipe[11], MIDAW[12], MARS[13], RACE[14], WebArray[15], EzArray[16], and Expression Profiler/Array Express[17]. These non-commercial options are viable solutions for some users and have greatly increased the ease in which microarray data analysis can be accomplished. However, none of these options encompasses a total solution that includes documenting the processing and quality assurance steps in the array generation process, as well as providing a means of storing, analyzing, and sharing microarray data specific to the Agilent platform.

To address the problems of dealing with microarray data generated on the Agilent DNA microarray platform, we have developed a software tool, EDGE^3^, an open-source, freely available, web-based software application that allows for the storage, analysis, and controlled sharing of transcription-based microarray data generated on the Agilent DNA platform. In addition, EDGE^3 ^also provides a means for a lab or genomics core to manage RNA samples and arrays during the hybridization process via a well-defined workflow that aims to aid in meeting or exceeding MIAME guidelines[18]. EDGE^3 ^has been designed with the assumption that methods for background correction and normalization have been designated within the Agilent Feature Extraction software package. Subsequently, the signal intensities and log ratios have been calculated using Agilent Feature Extraction software-based algorithms.

EDGE^3 ^is freely available for download at  with the source code distributed via a Subversion (SVN)[19] repository or a compressed archive.

## Implementation

**Figure 1:**
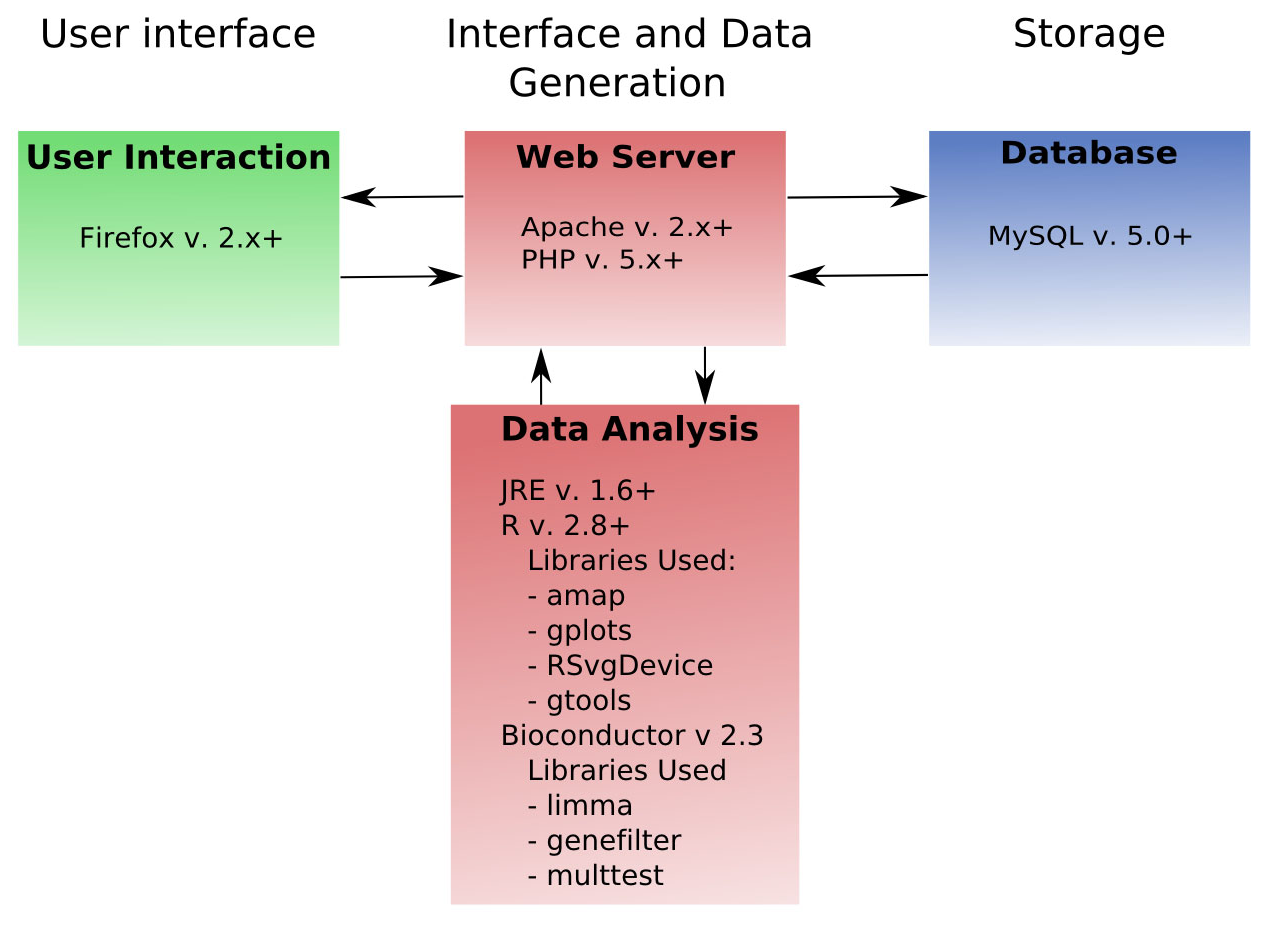
**The three layers of EDGE^3 ^composed of four software components**. Communication between the User Interface layer and the Interface and Data Generation layer takes place between the User Interaction component (i.e., the web browser) and Web Server component. Queries from the User Interface layer are routed through the Web Server component to the Storage layer using SQL. Data is returned from the Database component to the Web Server Component and, if necessary, routed to the Data Analysis component where the bioinformatics-based computations take place. If required, the results from the Data Analysis component are returned to the Web Server. In all cases, requested information is sent to the end user and displayed in the User Interaction component (i.e., Firefox web browser) via the Web Server component.

Throughout the development process we have relied on a number of open-source software solutions. The software of EDGE^3 ^can be thought of as consisting of four components separated into 3 layers of integration: a User Interface layer, an Interface and Data Generation layer, and a Storage layer (Figure 1). The first component, User Interaction, is the interactive, front-end interface and comprises the User Interface layer. To access EDGE^3^, the only software required of the end-user is an internet browser. We suggest utilizing Firefox version 3.0+ as it conforms to a high degree with open standards and is available for use on most operating systems. The Interface and Data Generation layer is composed of two components: a Web Server component and a Data Analysis component. The Web Server component of EDGE^3 ^is the software utilized to generate the front-end interface. The generation of this front-end is accomplished by Apache web server with PHP. EDGE^3 ^has been tested extensively with Apache version 2.2.8 and PHP 5.2.4-2. The Data Analysis component encompasses the software utilized to perform the various data analysis functions. To that end, Java version 1.6+ of the runtime compiler and the R statistical package version 2.8+ [20] and Bioconductor[21] are required for aspects of data analysis.

The Storage layer is composed of the Database component. The Database component is the back-end relational database used for the storage of microarray data and associated information. MySQL version 5.0.5 is utilized as the back-end relational database. The database schema currently consists of 41 tables. See additional file 1: EDGE^3 ^database schema. The MySQL database server is not necessarily required, as the database schema of EDGE^3 ^could be utilized with other database engines due to the use of the ADOdb Database Abstraction Library for PHP[22].

## Results

### Two major functions of EDGE^3^

EDGE^3 ^has been developed with two major functions in mind. The first function is to provide a means to manage and annotate microarray-based experiments utilizing a workflow for the generation of microarray data. This function is an administrative one and its implementation involved the development of a basic information management system to track the progress of user-submitted RNA samples to our microarray facility for processing. The second function of EDGE^3 ^is to allow for easy storage, analysis, and sharing of microarray data in a manner that is simple, accessible, and conducive to collaboration. The majority of the development of EDGE^3 ^has been done within the context of integration with the Agilent two color platform. From the standpoint of an end-user, the two different functions can be thought of as an experiment management section and an experiment data analysis section, respectively. In reality, the experiment management section can be decoupled from the data analysis section and serve as a means to archive array data and monitor quality control. However, the data analysis section is dependent on the back-end database and the server file system.

### Experiment Management User Interface

**Figure 2:**
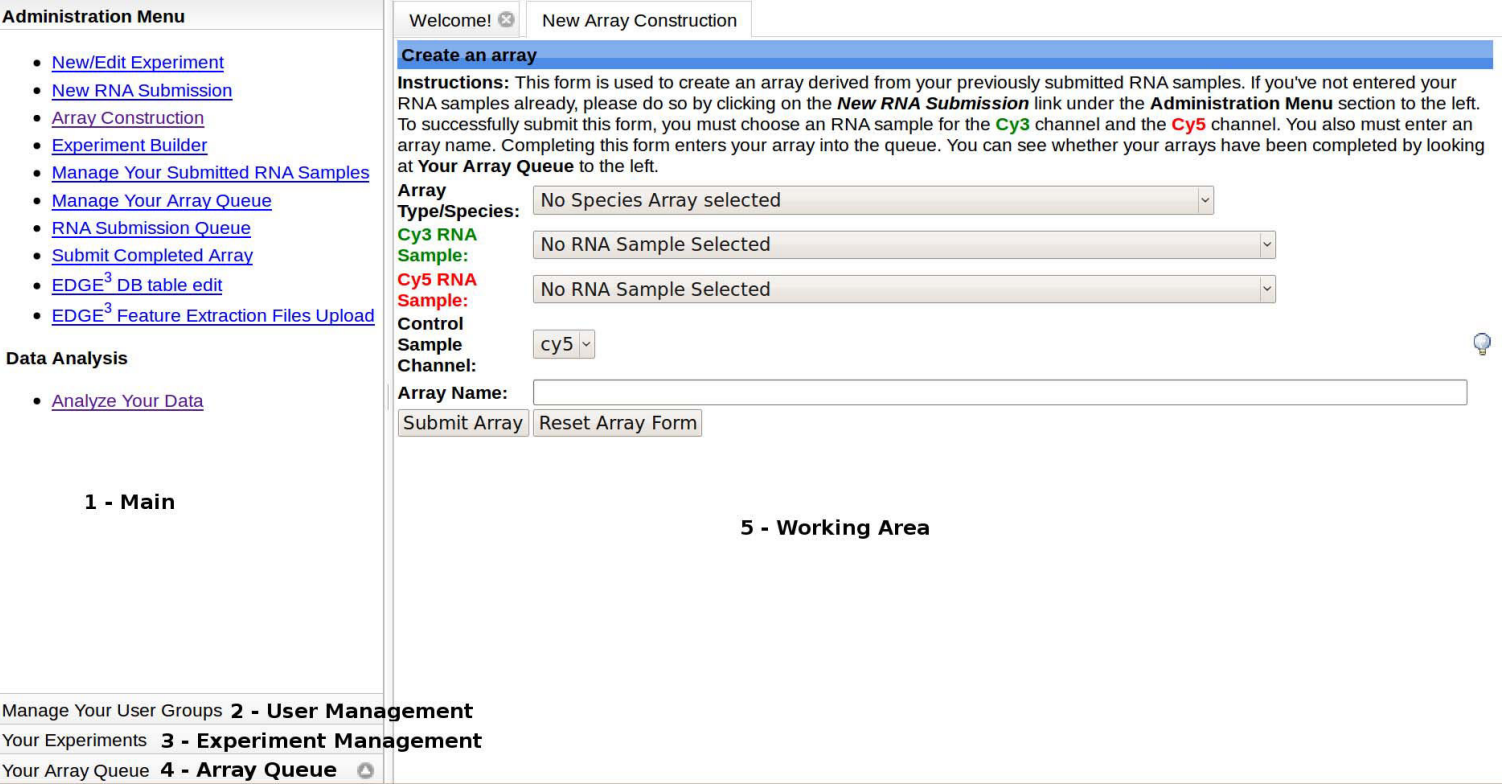
**Experiment Management User Interface**. This figure represents an example of what the end-user views within the Experiment Management Section. There are five enumerated sections with numbers 1 through 5. The left hand side of the screen containing sections 1-4 is composed of what is called an accordion pane. Clicking on the individually labelled pane headers will expand their contents for viewing. The first section, 1 - Main, is located in the upper left hand side of the screen. This is where you can select what function of the Experiment Management Section you wish to utilize to construct your experiments. The second section, 2 - User Management, is located below the Main Menu section. This section allows for the creation of user groups and the assignment of groups to experiments the end-user owns or administers. The third section, 3 - Experiment Management, lists all the experiments the end-user owns or is associated with via group membership. Here the end user can download data and quality control files. The fourth section, 4 - Array Queue, is where the status of an end-user's submitted arrays are displayed. (See below for details on Experiments and Arrays.) The fourth section, 5 - Working Area, is the section where the majority of end-user input takes place.

The Experiment Management User Interface is composed of five main sections. The layout of the interface is intended to provide an intuitive and simple means to manage and monitor the progress of a microarray experiment created in EDGE^3^. Figure 2 gives a brief explanation of each section.

### EDGE^3 ^experiment-based object hierarchy

Management of array processing utilizes three fundamental objects: 1) Experiment, 2) Array and 3) RNA Sample. See additional file 2: Three main objects in EDGE3 Experiment Management. When an object is created, it is assigned a unique identifier allowing for the association with descriptive information and data. An Experiment object is created to contain Array objects. An Array object can be a part of any number of experiments. An Array object is created to contain RNA Sample objects. As an example, a two-channel Array object consists of two RNA samples, one for the Cy3 channel and one for the Cy5 channel. What makes an Array object unique is the hybridization instance of its RNA Sample object components. A hybridization instance in this case can be thought of as a single instance of one RNA sample hybridized with another RNA sample on one distinct array. Thus, technical replicates using the same RNA Samples labelled in the same or opposite manner are treated as unique arrays. An RNA Sample object is the most fundamental object in the experiment-based object hierarchy of EDGE^3^. Since RNA Sample objects are able to be associated with multiple Array objects and, possibly, multiple experiments, RNA Sample objects require the most detailed information.

### Conforming to MIAME Guidelines

Utilization of the three objects composing the EDGE^3 ^experiment-based hierarchy aims to provide a means to satisfy the objective of meeting MIAME guidelines. Though the objects provide direction towards that objective, without adequate curation it is difficult to ensure the adherence to MIAME standards. Initially, the onus of MIAME adherence falls on the end-user submitting RNA samples. To facilitate compliance, error checking features during submission help to ensure that a detailed level of information is provided. However, array processing staff can review submissions and suggest changes that would aid in meeting annotation objectives. At the level of RNA Samples objects, EDGE^3 ^is most stringent in its requirements for detailed information. This is due to the fact that the RNA samples are the most important part of the assay. Accurate information regarding the quality and source of the RNA are paramount in determining whether or not the data generated are of any value, regardless of whether or not the hybridization process appears to be successful. EDGE^3 ^provides the ability to store image or text files associated with the assessment of the quality and amount of RNA within the context of an RNA Sample object. This information is also important for any RNA samples that may be stored for later studies or repeated analyses (i.e., technical replicates). Descriptive information required for RNA Samples include sample name, the various environmental conditions or exposures the originating tissue sample was subjected to, the organism the sample was derived from, the tissue/cell type of origin, etc.

At the level of Array objects, each array image, data files, and the associated quality control files generated by Agilent Feature Extraction Software are associated with their respective Array objects and archived within the file system of EDGE^3^. The image and quality control files can subsequently be retrieved and assessed by the end-user to judge the quality of the hybridization results. See additional file 3: Data and Quality Control.

At the level of an Experiment object, information including the purpose or hypothesis under consideration and the experimental design are required. Experiments represent the synthesis of the Array and RNA samples and the descriptive information associated with an experiment should be adequately represented.

### EDGE^3 ^Administration Workflow

**Figure 3:**
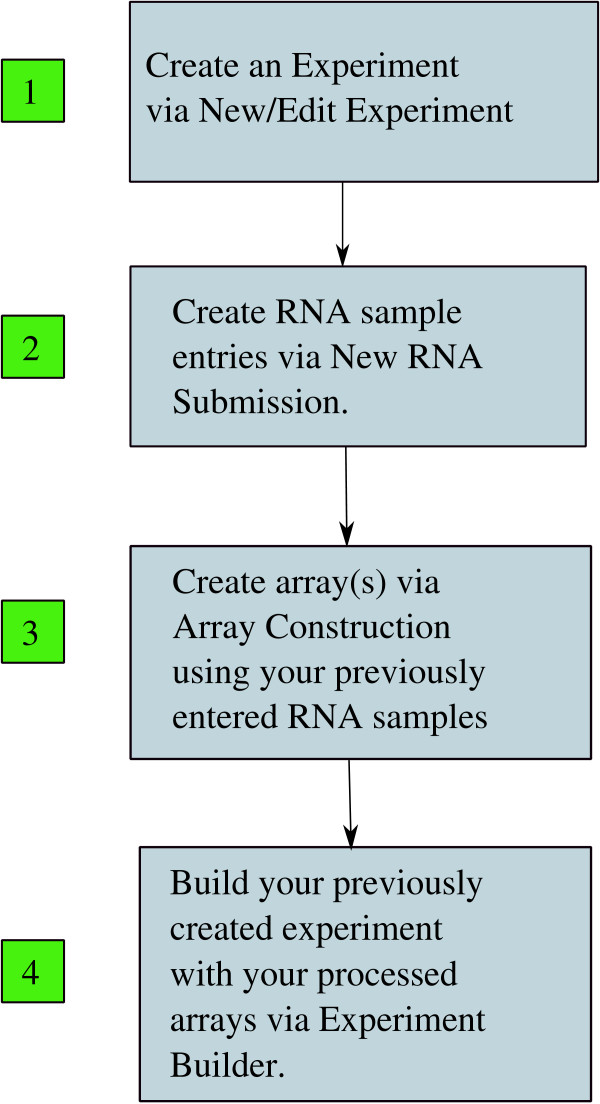
**EDGE^3 ^Workflow**.

**Figure 4:**
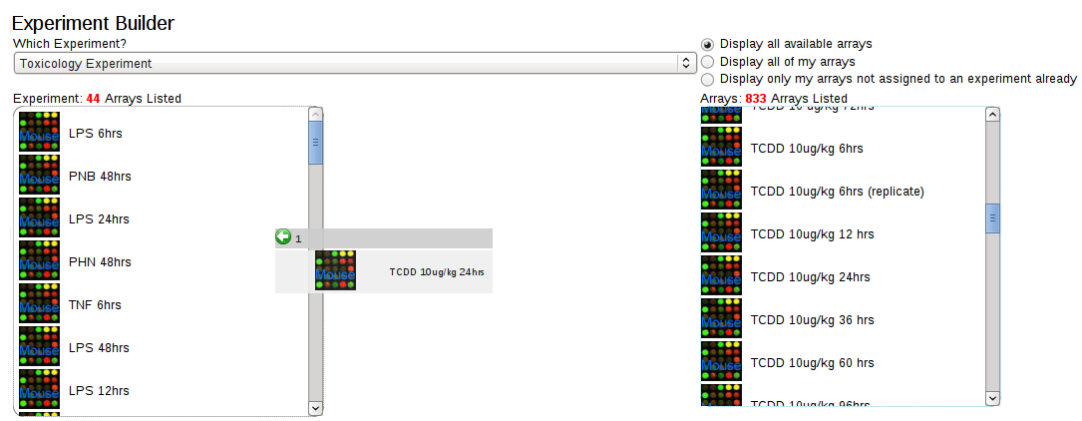
**The Experiment Builder**. This screen shot depicts the addition of a processed array to an end-users predefined experiment. Like most user interfaces the layout is simple and easy to utilize. In this case an intuitive 'drag-and-drop' functionality is utilized to add or remove arrays to/from a selected experiment. The arrays are represented their user-assigned name and icons labeled with their respective organism source.

The culmination of the object hierarchy coupled with the requirement of associated quality assurance data and experimental information resulted in the establishment of a microarray workflow. There are four main steps in the EDGE^3 ^Administration Workflow (Figure 3). For an end-user to progress through the workflow, a series of forms are utilized to enter information and subsequently parse the information entered for errors and omissions. The workflow allows for tracking the status of arrays created and placed in the processing queue by end-users. During the workflow process, creation of an RNA sample or an array by an end-user results in an email notification to the array processing staff to alert them of the impending arrival of RNA samples. When an array has been completely processed the end-user is notified by email and at that point the end-user can assess the quality of the array hybridization. After the arrays are approved by the end-user, they can then associate the completed array(s) with an already created experiment (Figure 4) and begin to analyze the data in the context of their predefined experiment.

The process of generating microarray data has a number of quality assurance steps. It is generally assumed that prior to labelling RNA samples, the quality of the RNA is assessed by either gel electrophoresis or a microfluidics-based instrument such as the Agilent Bioanalyzer 2100. Additionally, in the case of Agilent two-color arrays, measurement of the yield and specific activity after a reverse transcription-based labelling step is done to ensure successful labelling. It could be argued that the documentation of quality assurance steps such as these could be done utilizing traditional methods of documenting experiments such as the laboratory notebook. However, the need for computational-based resources to store, analyze, and share data suggests association of the quality assurance steps with the data for easy referencing when data quality questions arise. To this end, EDGE^3 ^has the capability to associate these quality control data with microarrays during the processing steps allowing for a great deal of transparency in assessing the quality of data generated.

### Storing microarray data

Microarray data are stored in two ways within EDGE^3^. First, all files generated by the Agilent Feature Extraction software are archived in the file system of the server EDGE^3 ^is installed on. If necessary, the data files can be compressed to conserve hard disk space. These files are readily available for download in compressed format by authorized users/owners of the data. Second, the files containing the feature extracted data are imported into a back-end relational database offering the ability for efficient querying during data analysis.

### Data Analysis User Interface

**Figure 5:**
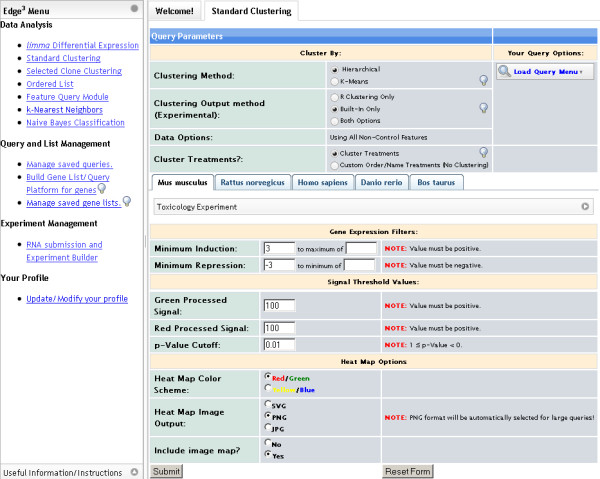
**Data Analysis Interface**. A representation of the Data Analysis Interface after logging in and choosing Standard Clustering from the menu option under the Data Analysis subsection. This section is divided vertically into two sections. The column on the left-hand side contains various menu options and links to instructions. The section on the right contains the working area where queries are generated and data analysis takes place. In general, the layout remains consistent when performing different functions.

The Data Analysis User Interface is very similar to the Experiment Management User Interface. The Data Analysis User Interface consists of three main sections designed to provide an intuitive and clean user interface. Figure 5 depicts the layout of the interface.

### Data Analysis Objects

Two objects are associated with the Data Analysis Interface, the Query Object and the Gene List Object. A Query Object is built utilizing a series of HTML forms to enter the parameters required for the particular data analysis module chosen. When all of the query parameters for a selected module are entered the query is submitted for generation of the results. After a query has been completed and the results returned a temporary Query Object is created in the database. The end-user has the option to save this query for future use. Once a query has been saved to the database, the end-user can then recall the query and either reissue the query without having to re-enter any of the parameters or modify the query's existing parameters. If changes have been made to a previously saved query the end-user has the option to update the query with the new changes or save the modified query as a new query.

The Gene List Object is used in a couple of ways. First, a Gene List Object can be used to store the lists of genes generated from a query result set. Second, an end-user has the ability to build their own gene lists based on a number of criteria. The first method of utilization is based on the idea that a large number of microarray experiments are performed with the goal to obtain a set of differentially expressed genes between one or more groups. Saving a gene list in this instance allows the end-user to generate new queries with the gene list in the Selected Clustering Module or the Ordered List Module. These modules take a gene list as one of their input parameters and, instead of querying the entire set of probes printed on an array, apply any filtering criteria to that distinct set of genes. The second method of utilization allows the end-user to build a custom list of genes based on a number of specified criteria including Official Gene Symbol, Refseq and GO terms. The end-user now has the ability to use their custom set of genes as an input parameter to the Selected Clustering Module or the Ordered List Module.

### Data Analysis Methods implemented

EDGE^3 ^has a number of built-in algorithms for microarray analysis and offers additional means of analysis via integration with R/Bioconductor. The built-in algorithms are separated into different modules utilizing algorithms including unsupervised methods such as k-Means clustering and hierarchical clustering and supervised methods such as k-Nearest Neighbors classification, similarity queries[23], and Naive Bayes classification. Basic statistical methods such as Student's t-Test and ANOVA can be utilized to identify differentially expressed genes with correction methods to account for the multiple testing problem. The built-in algorithms have been primarily designed to identify differentially expressed genes in experiments using a reference design.

Some of the benefits of the built-in algorithms include a higher degree of interactivity with the results such as linking to external databases (e.g., MGI, NCBI, etc.) and greater integration with the back-end database for easy access to quality control information and annotation. See additional file 4: Identifying and Clustering Differentially Expressed Genes.

EDGE^3 ^has been integrated with R/Bioconductor to provide the ability for analyzing data with more robust statistical methods. The Limma package is utilized to identify differentially expressed genes based on a moderated t-statistic and incorporation of empirical Bayes methods to borrow information between genes[24]. The Limma package offers the flexibility to take into consideration multi-factor experimental designs and time course experiments when trying to elucidate differentially expressed genes. Ancillary R/Bioconductor packages are utilized to visualize the data and generate interactive result sets. Benefits of using the R/Bioconductor algorithms include a wider variety of algorithm choices and input parameters. Additionally, in some cases, results are returned faster. See additional file 5: Identifying differentially expressed genes using Limma.

### Data Sharing via User Access Control

Although EDGE^3 ^can be implemented as entirely open system it has a built-in user access control system. This control system is based on the two objects, Users and Groups. User objects are individual researchers registered within the database. Group objects are composed of User Objects. This structure allows for both collaboration and a moderate level of access control. Users can create Group objects and become the administrator of the Group they create. Group administrators can add Users to a Group they created and share administration rights by assigning added users as administrators. Access rights to Groups are granted at the level of Experiment Objects allowing for access to Array Objects and RNA Sample objects that compose the experiment.

## Discussion

EDGE^3 ^has evolved from a previous iteration used to store, analyze and share data generated on a custom cDNA array[25]. EDGE^3 ^has been developed with the intention to capture as much information as possible during the Agilent array processing workflow and to take the data generated by the Feature Extraction platform software and make it amenable to efficient and effective storage, analysis, and sharing. These combined features help to set EDGE^3 ^apart from other web-based microarray programs as well as most stand-alone commercial and non-commercial applications.

To aid the end-user, an extensive set of instructions is available. Instructions detailing step-by-step written descriptions of the various data analysis methods are accessible via the 'Welcome' page. Additionally, a number of tutorials for the data analysis methods are available in Adobe Flash format.

Comparing EDGE^3 ^to currently available web-based microarray analysis allows for an understanding of how EDGE^3 ^could be a viable option for labs or microarray facilities that conduct collaborative research with the Agilent two-color microarray platform. See additional file 6: Features comparison. One of the main differences between EDGE^3 ^and a majority of currently available software packages is its integration of the entire microarray workflow process from the experimental planning and annotation stage to the point of data analysis and data sharing among collaborators.

### Future Directions

Though, in it its current state EDGE^3 ^provides a powerful and coherent web-based software tool to manage the Agilent array workflow process there are some features that would further enhance its utility. To that end, we plan on developing the EDGE^3 ^software to include the ability to easily import data generated on the Agilent platform from large microarray repositories such as the Gene Expression Omnibus[26] and ArrayExpress[27]. Additionally, EDGE^3 ^is focused on two-channel microarray expression data, but we intend to extend the functionality to include data generated by utilizing single channel arrays.

Currently, the user access control model used is fairly rudimentary and could be further improved by implementing a hierarchical structure where groups can be members of other groups. EDGE^3 ^was developed in a setting where the security of data was not of paramount interest. In a clinical setting where patient data are being stored and analyzed it would be best to implement some form of encryption such as Secure Socket Layers. This is a feature that could be implemented at the level of the web server.

## Conclusion

In summary, EDGE^3 ^is an open-source, web-based application that allows for the storage, analysis, and controlled sharing of transcription-based microarray data generated on the Agilent DNA platform. In addition, EDGE^3 ^provides a means for managing RNA samples and arrays during the hybridization process with the goal of adhering to MIAME guidelines. EDGE^3 ^accomplishes this through the utilization of open-source software and an intuitive user interface. EDGE^3 ^is a viable option for microarray facilities or research laboratories who are utilizing the Agilent array platform.

## Availability and requirements

**Project name**: EDGE^3^

**Project home page**:  with source code hosted at  in a SVN repository.

**Operating system**: For the end-user elements, any operating system that can run Mozilla Firefox 2.0+. For the server elements, any operating system that can run Apache 2.x+ with PHP 5.x+, JRE v1.6+, R v. 2.7.2, and MySQL v. 5.0+ (or compatible database server).

**Disk Space Requirements**: Estimated space requirements are as follows: 63-140 Megabytes (MB) for EDGE3 web server files, 100 MB for default install of EDGE3 database without array data, 20 MB for each array in the database, and 114 MB for the Feature Extraction files per array.

**Programming Language**: Java, HTML, PHP, R, Javascript

**Other Requirements**: Server with at least 2 GB of memory.

**License**: GNU General Public License

**Any Restrictions to use by non-academics**: None

**Anonymous review of EDGE^3^**: The data analysis aspects of EDGE^3 ^can be accessed at  without having to install locally. The guest user account (Username/password: guest/guest) provides access to one experiment consisting of 15 microarrays.

## Authors' contributions

ALV and CAB developed the main idea. ALV drafted the manuscript, created the associated database, and coded the majority of EDGE^3^. AAS and MC developed portions of the EDGE^3 ^software and helped to direct the writing of the manuscript. All authors read and approved the final version of the manuscript.

## Supplementary Material

Additional file 1 **EDGE^3 ^database schema**. This figure represents the table structure of the EDGE^3 ^database. Information displayed includes the fields within the tables and their respective types, the primary key(s) for each table (identified by 'key' icon), the indexes associated with each table, how the tables are interconnected, and how the conceptual objects (RNA sample object, Array object, etc.) are defined/composed. Click here for file

Additional file 2 **Three main objects in EDGE^3 ^Experiment Management**. An example of the EDGE^3 ^object hierarchy in the form of an Experiment composed of six Arrays and seven RNA Samples. This Experiment utilizes a reference RNA Sample, RNA Sample C, and six non-Reference samples. An Experiment can be composed of any number of Arrays and an Array is composed of two RNA samples. Though arrays must be unique, they do not have to be derived from unique RNA samples. The red RNA samples in this figure represent the Cy5 labelled samples and the green RNA samples represent the Cy3 labelled samples. Click here for file

Additional file 3 **Data and Quality Control**. (A) For each experiment, the end user has the ability to download all of the raw data files of the associated arrays. The files are compressed to expedite the download process. (B) A screenshot of the Array/Info Edit function. Here the end user can download data and quality control files associated with an array. (C) For each individual array within an experiment the end-user owns or is associated with, the JPG image generated by Feature Extraction Software generated is available for viewing and download. (D) An example of part of a quality control file generated by the Feature Extraction Software allowing end-users to assess the quality of the array hybridization process. Click here for file

Additional file 4 **Identifying and Clustering Differentially Expressed Genes**. A series of screenshots to identify differentially expressed genes between two groups (a control and a treated group with three biological replicates in each group) hybridized against a common reference sample. A) A screenshot of the Standard Clustering Module with the desired parameters established for the six arrays chosen. (B) A screenshot of the form used to order or, as in this case, to group the arrays based on the number of groups chosen on the previous form. The groups are assigned by the end-user with Corn Oil control biological designated, "Control", and the TCDD-treated biological replicates assigned to group "2". Because the purpose of this query is to identify differentially expressed genes between two groups, we've chosen to do a t-Test at a specified alpha of 0.001 with rough False Discovery Rate correction. (C) This is a screenshot of the results page. The 59 probes determined to be differentially expressed based on the parameters of the query have been hierarchically clustered. From the results page the end user can obtain the associated data files including fold-change values, the processed signal values of the cy3 and cy5 channels for all of the returned probes, and the ordered p-Values of the probes meeting the criteria chosen. Additionally, the resulting heatmap is available in different output formats including scalable vector graphics (SVG), PNG, and JPG. The end user has the option to make the map "hot-clickable" such that each spot, gene name, and array name can be clicked on to obtain quality control information and annotation information. This query can also be saved for reuse or modification so the end-user doesn't have to re-enter the parameters. (D) A portion of a results table displaying 20 of genes designated as being differentially expressed. Click here for file

Additional file 5 **Identifying differentially expressed genes using Limma**. A series of screenshots giving examples of output where Limma has been utilized to identify differentially expressed genes using the same two groups of arrays as those in Additional file 4. (A) A list of differentially expressed genes where the calculated p-Value of the moderated t-statistic is less than 0.001. There were eighty-one genes that met the criteria. (B) A volcano plot representation of the distribution of the differentially expressed genes. Click here for file

Additional file 6 **Features Comparison**. A table comparing the features of EDGE^3 ^with other non-commercial microarray analysis tools. Click here for file
