# Supplementary figures and images for: EDGE3: A web-based solution for management and analysis of Agilent two color microarray experiments
Source: BMC Bioinformatics. 2009 Sep 4;10:280. doi: 10.1186/1471-2105-10-280 (PMC2746223; doi:10.1186/1471-2105-10-280)

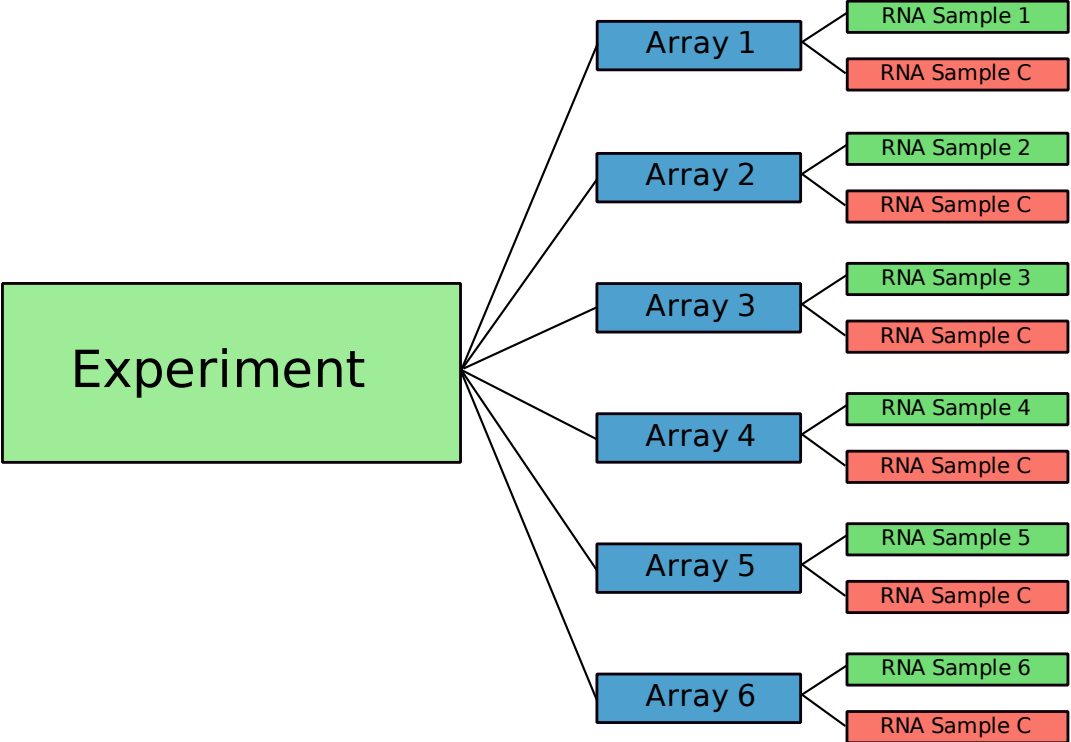

Supplement: Additional file 2 — Three main objects in EDGE3 Experiment Management. An example of the EDGE3 object hierarchy in the form of an Experiment composed of six Arrays and seven RNA Samples. This Experiment utilizes a reference RNA Sample, RNA Sample C, and six non-Reference samples. An Experiment can be composed of any number of Arrays and an Array is composed of two RNA samples. Though arrays must be unique, they do not have to be derived from unique RNA samples. The red RNA samples in this figure represent the Cy5 labelled samples and the green RNA samples represent the Cy3 labelled samples. [file 1471-2105-10-280-S2.pdf]

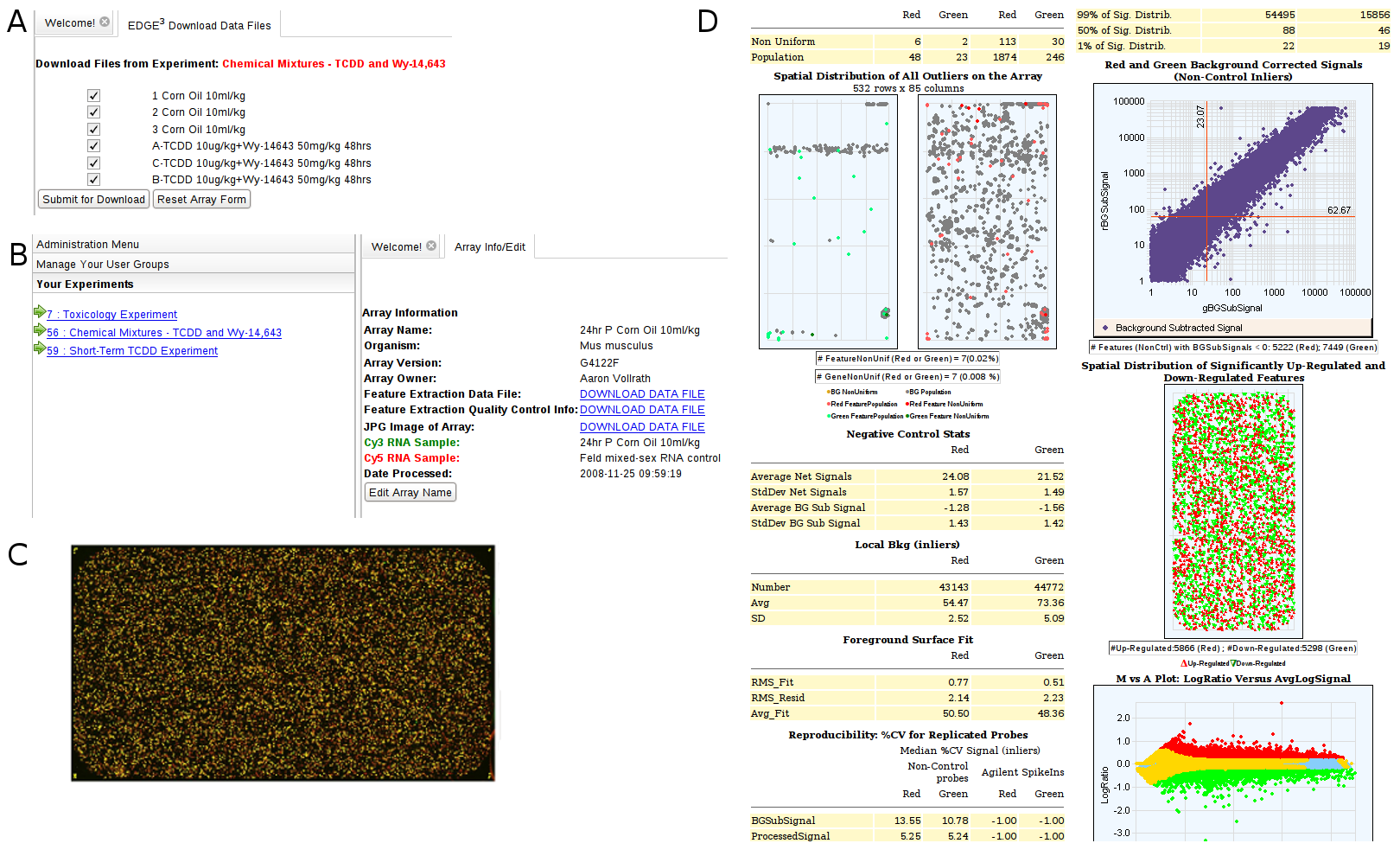

Supplement: Additional file 3 — Data and Quality Control. (A) For each experiment, the end user has the ability to download all of the raw data files of the associated arrays. The files are compressed to expedite the download process. (B) A screenshot of the Array/Info Edit function. Here the end user can download data and quality control files associated with an array. (C) For each individual array within an experiment the end-user owns or is associated with, the JPG image generated by Feature Extraction Software generated is available for viewing and download. (D) An example of part of a quality control file generated by the Feature Extraction Software allowing end-users to assess the quality of the array hybridization process. [file 1471-2105-10-280-S3.png]

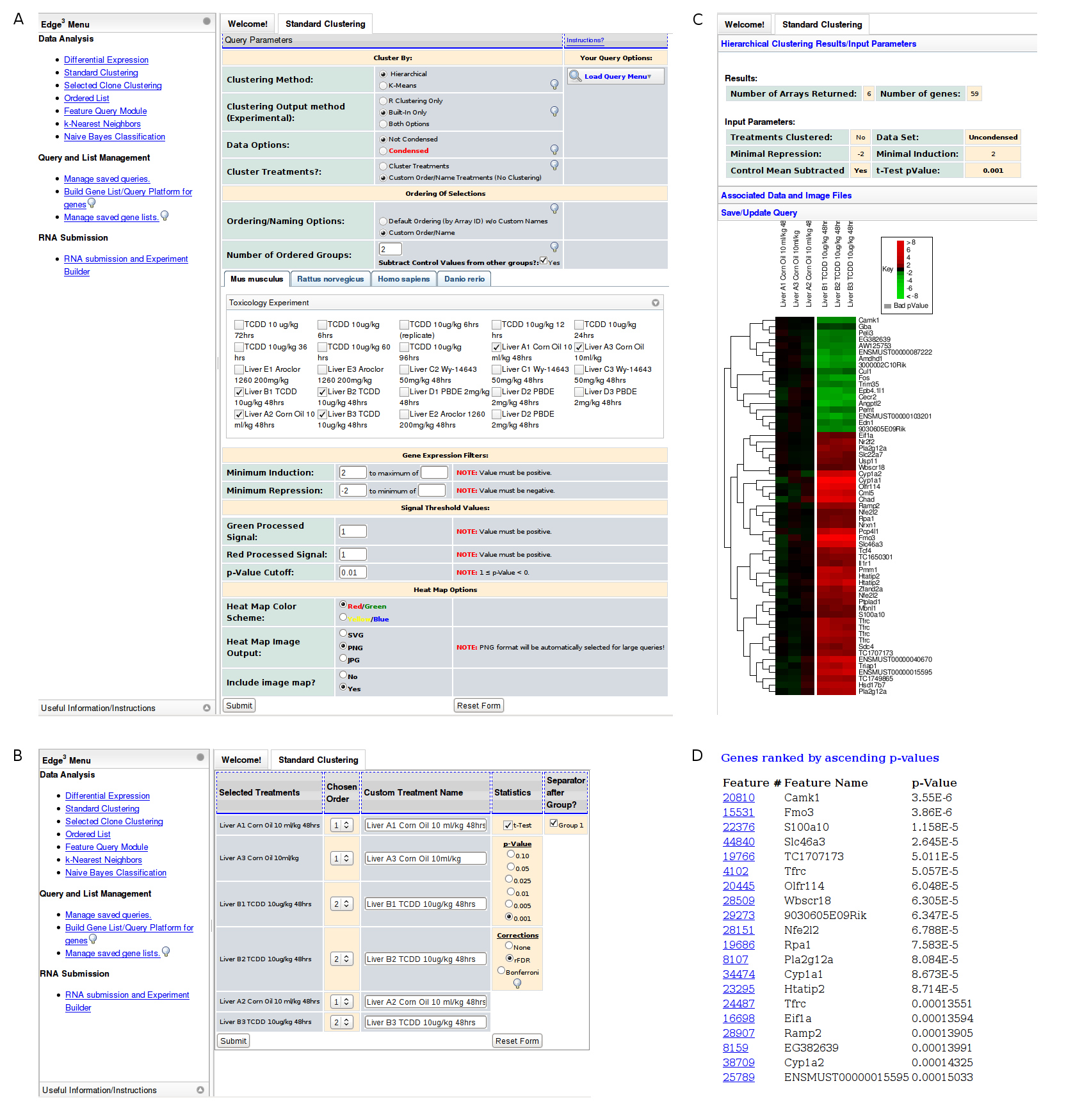

Supplement: Additional file 4 — Identifying and Clustering Differentially Expressed Genes. A series of screenshots to identify differentially expressed genes between two groups (a control and a treated group with three biological replicates in each group) hybridized against a common reference sample. A) A screenshot of the Standard Clustering Module with the desired parameters established for the six arrays chosen. (B) A screenshot of the form used to order or, as in this case, to group the arrays based on the number of groups chosen on the previous form. The groups are assigned by the end-user with Corn Oil control biological designated, "Control", and the TCDD-treated biological replicates assigned to group "2". Because the purpose of this query is to identify differentially expressed genes between two groups, we've chosen to do a t-Test at a specified alpha of 0.001 with rough False Discovery Rate correction. (C) This is a screenshot of the results page. The 59 probes determined to be differentially expressed based on the parameters of the query have been hierarchically clustered. From the results page the end user can obtain the associated data files including fold-change values, the processed signal values of the cy3 and cy5 channels for all of the returned probes, and the ordered p-Values of the probes meeting the criteria chosen. Additionally, the resulting heatmap is available in different output formats including scalable vector graphics (SVG), PNG, and JPG. The end user has the option to make the map "hot-clickable" such that each spot, gene name, and array name can be clicked on to obtain quality control information and annotation information. This query can also be saved for reuse or modification so the end-user doesn't have to re-enter the parameters. (D) A portion of a results table displaying 20 of genes designated as being differentially expressed. [file 1471-2105-10-280-S4.png]

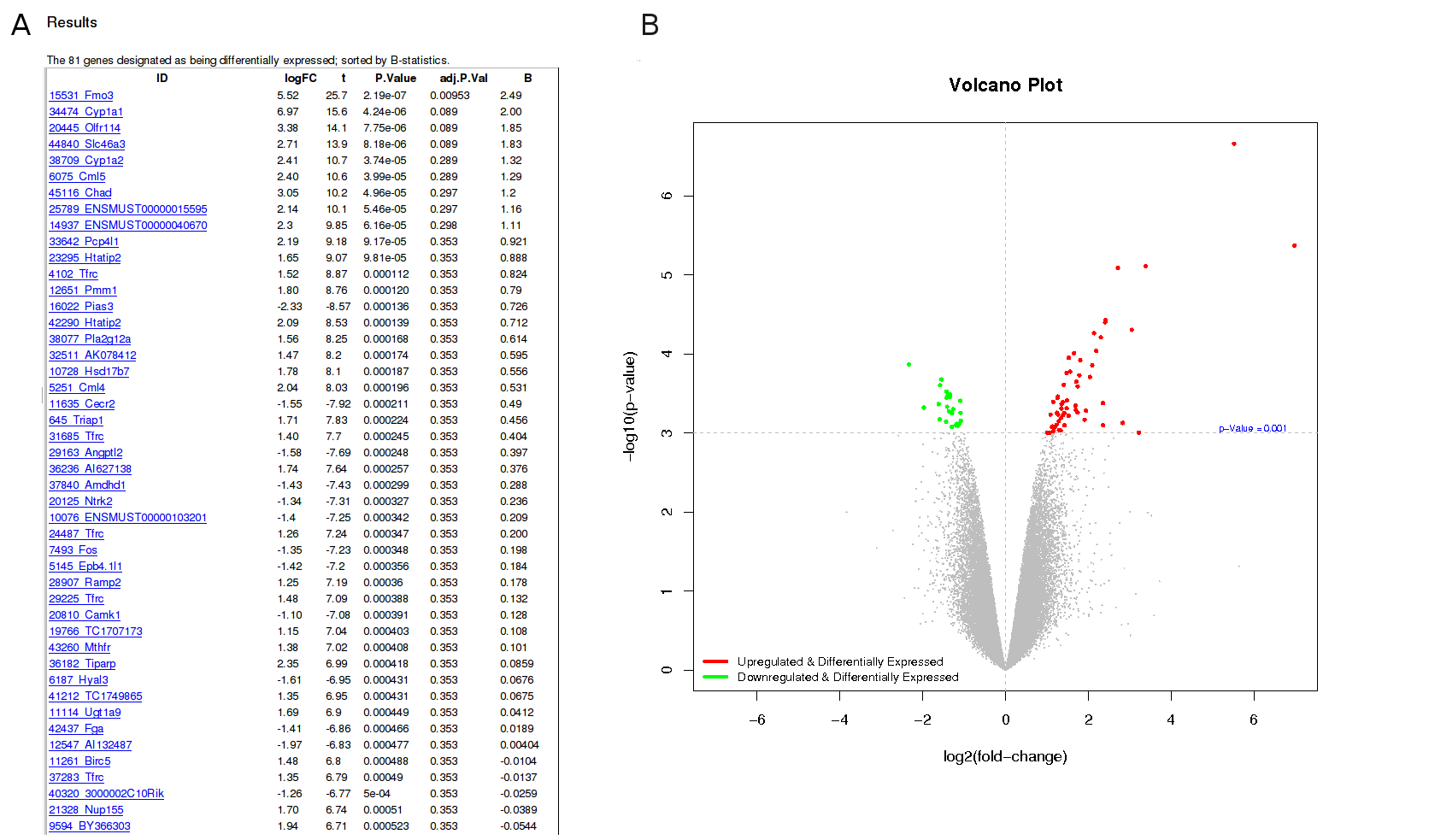

Supplement: Additional file 5 — Identifying differentially expressed genes using Limma. A series of screenshots giving examples of output where Limma has been utilized to identify differentially expressed genes using the same two groups of arrays as those in Additional file 4. (A) A list of differentially expressed genes where the calculated p-Value of the moderated t-statistic is less than 0.001. There were eighty-one genes that met the criteria. (B) A volcano plot representation of the distribution of the differentially expressed genes. [file 1471-2105-10-280-S5.png]
