# Supplementary material for: EDGE3: A web-based solution for management and analysis of Agilent two color microarray experiments
Source: BMC Bioinformatics. 2009 Sep 4;10:280. doi: 10.1186/1471-2105-10-280 (PMC2746223; doi:10.1186/1471-2105-10-280)
Supplement: Additional file 1 — EDGE3 database schema. This figure represents the table structure of the EDGE3 database. Information displayed includes the fields within the tables and their respective types, the primary key(s) for each table (identified by 'key' icon), the indexes associated with each table, how the tables are interconnected, and how the conceptual objects (RNA sample object, Array object, etc.) are defined/composed. [file 1471-2105-10-280-S1.pdf]

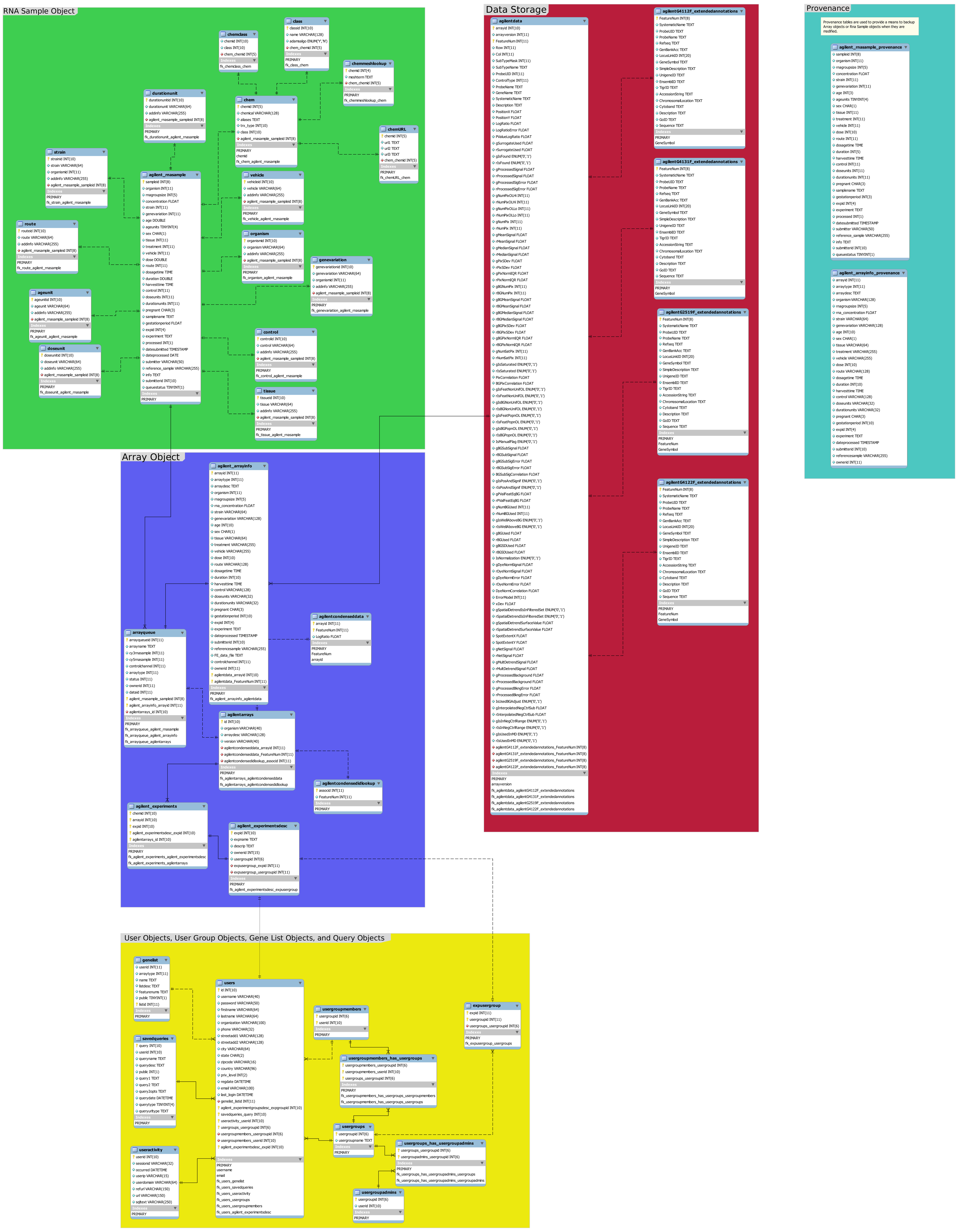
RNA Sample Object
Data Storage
Provenance
Array Object
User Objects, User Group Objects, Gene List Objects, and Query Objects
